# Microwave Study of Field-Effect Devices Based on Graphene/Aluminum Nitride/Graphene Structures

**DOI:** 10.1038/srep44202

**Published:** 2017-03-09

**Authors:** Mohammad Adabi, Johannes Lischner, Stephen M. Hanham, Andrei P. Mihai, Olena Shaforost, Rui Wang, Ling Hao, Peter K. Petrov, Norbert Klein

**Affiliations:** 1 Department of Materials, Imperial College London, London SW7 2AZ, UK; 2 National Physical Laboratory, Teddington, Middlesex, TW11 0LW, UK

## Abstract

Metallic gate electrodes are often employed to control the conductivity of graphene based field effect devices. The lack of transparency of such electrodes in many optical applications is a key limiting factor. We demonstrate a working concept of a double layer graphene field effect device that utilizes a thin film of sputtered aluminum nitride as dielectric gate material. For this system, we show that the graphene resistance can be modified by a voltage between the two graphene layers. We study how a second gate voltage applied to the silicon back gate modifies the measured microwave transport data at around 8.7 GHz. As confirmed by numerical simulations based on the Boltzmann equation, this system resembles a parallel circuit of two graphene layers with different intrinsic doping levels. The obtained experimental results indicate that the graphene-aluminum nitride-graphene device concept presents a promising technology platform for terahertz- to- optical devices as well as radio-frequency acoustic devices where piezoelectricity in aluminum nitride can also be exploited.

Graphene is a promising material for terahertz-to-optical devices, in particular voltage controlled attenuators and modulators for terahertz components to be used for wireless communication with high data rate^1^,^2^. The utilization of the field-effect as well as the ultrafast carrier dynamics in graphene devices that are fabricated by wafer-scale chemical vapor deposition coupled with the matured transfer technique onto arbitrary substrates transform this 2D material into a promising choice for the realization of ultrafast wireless communication devices and sensors^3^. The ambipolar field effect is one of the unique features of graphene, which allows the control of the carrier concentration by an externally applied electric field^4^. At microwave frequencies, intraband transitions provide the dominant contribution to the conductivity resulting in a mainly resistive response with a nearly frequency-independent real-valued surface impedance up to frequencies of about 100 GHz^5^,^6^,^7^. Operation of a variety of devices, including tunable antennae^8^, optical modulators and waveguides^9^,^10^,^11^,^12^,^13^,^14^, amplifiers^15^ and broadband terahertz modulators^16^ has already been demonstrated. However, using graphene itself as gate electrode of a graphene FET device allows to fabricate optical transparent devices, in case of AlN as a highly elastic gate dielectric even flexible FET devices. As an example, a transparent graphene FET based biosensor would enable *in-situ* optical microscopy of biological cells. Flexible and transparent FET devices may lead to novel device integration concepts such as wearable electronics and solar cells, integrated RFID tags in packaging material, and would therefore contribute to the ongoing electronic revolution^17^.

We realized a proof of concept for a self-gating graphene structure composed of an ultrathin dielectric aluminum nitride (AlN) layer sandwiched between two graphene layers. Previous attempts to fabricate double-graphene structures include implementation of poly (methyl methacrylate), PMMA, in between two graphene layers^18^. While fabrication of such structure is straightforward, PMMA suffers from retaining structural integrity in post-transfer processes. Moreover, the low breakdown voltages in polymers limits the range of achievable carrier densities. As illustrated in Fig. 1, the double graphene FET (DG-FET) structure is composed of a graphene-50 nm AlN -graphene stack on a HRS substrate with nSiO_2_ layer. The device is fabricated in three steps as demonstrated by Fig. 1. First (Fig. 1a), the bottom graphene layer (7 mm by 5 mm) is transferred onto nSiO_2_. This is followed by magnetron- sputtering of 50 nm AlN over the entire area at 400 °C substrate temperature (Fig. 1b) and then the top graphene layer (5 mm by 7 mm) is transferred over the AlN thin film (Fig. 1c). This arrangement was chosen to form an active area of 5 mm by 5 mm square that fully covers the testing aperture. This was achieved by carefully choosing the geometry of the copper substrate that was used in the graphene growth stage to avoid any photolithography/etching post-processes. The optical microscope image of the device reveals the expectedly clear contrast between the different layers as can be seen in Fig. 2c. The Raman spectrum as well as the 2D/G ratio distribution of graphene post AlN growth as shown in Fig. 2d strongly indicates that the sputtering process at 400 °C has not resulted in strong deterioration of graphene. The chosen growth temperature of AlN represents a compromise between the amount of crystallinity of the AlN layer and graphene quality, and is subject for future improvements (see Supporting information). As a contact-free method for graphene sheet resistance measurement, a dielectric-loaded microwave cavity technique that operates at approximately 8.7 GHz has been employed for this study^19^,^20^. Direct comparisons to DC results fully validates the accuracy of this robust approach (Supporting information S6). The graphene sample is placed on top of the aperture with the graphene-coated side facing upwards as illustrated in Fig. 2a.

The transverse electric, TE_01δ_, mode of the dielectric puck is the lowest fundamental mode that is excited via two coupling loop antennas in the cavity and the resulting resonance curve is measured using a Rohde-Schwarz ZVA vector network analyzer (VNA). This mode exhibits only azimuthal electric field component and therefore the field remains continuous on the surface of the ceramic resonator and high confinements of the field within the puck is achieved. The resulting evanescent field leaks through the aperture of the cavity and excites circular high-frequency currents in the graphene layers. As such, it represents an ideal measurement methodology for highly accurate and contact-free electrical measurements of 2D materials^20^. Figure 2b shows a CST Microwave Studio simulation result of the electric field penetrating through the aperture as well as the substrate. As shown in Fig. 2, we have designed the active area of the device such that the graphene layers fully cover the aperture and circular currents are excited via the evanescent field.

The entire GFET device is semi-transparent for the electromagnetic fields of the TE_01δ_ mode^21^,^22^. The electrical contacts are placed outside the area penetrated by the electromagnetic field and have no measurable influence on the cavity *Q*-factor. Prior to presenting the double-gated structure, we first verify this characterization technique on a simpler commonly employed single gated silicon based system. For this system, we transferred graphene on both nSiO_2_/Si and AlN/Si substrates. This was then followed by the microwave field-effect measurements using the dielectric-loaded cavity method. Figure 3a–c illustrate our procedure for converting microwave transmission measurements into the gate voltage dependence of the sheet resistance for a given G-FET device. The transmission characteristic measured by the VNA (Fig. 2a) is used to extract the quality factor at any given gate voltage (Fig. 3b) by an accurate fit, as described in a previous study^22^. Introduction of the graphene-covered substrate leads to a reduction of the resonator’s quality factor *Q*, which determines the sheet resistance via equation (1)^20^.


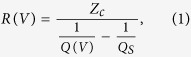


Equation (1) is employed in calculation of the sheet resistance at a given gate voltage via the measured *Q*-factor values. Here, the characteristic impedance of the cavity, *Z*_*C*_, is determined by a substrate calibration based on the measured shift of the resonant frequency due to the graphene-free substrate^19^. *Q*_S_ denotes the measured quality factor with the uncoated HRS substrate and *Q(V*) is the measured quality factor at a given gate voltage for a graphene covered substrate. The ratio between the resistance *R(V*) at a given gate voltage *V* (typically at the Dirac point) and the minimum resistance *R*_min_, (representing maximum field induced n- or p-type doping), given by equation (2).


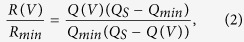


Here, *Q*_min_ represents the smallest measured quality factor corresponding to the minimum sheet resistance. Note that this ratio is independent of the cavity calibration factor *Z*_C_. It is important to note that the achieved modulation depth of 2.1 dB is much lower than the maximum achievable modulation depth for a graphene-modulated microwave cavity. This is merely because the silicon substrate, in spite of its high resistivity, contributes significantly to the cavity losses.

We compare the voltage dependent conductivity to the theoretical Boltzmann equation results given by equation (3)^23^:


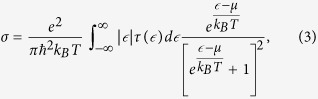


where *k*_*B*_ and *μ* denote the Boltzmann constant and the chemical potential, respectively, *T* the temperature, and 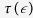 is an energy-dependent scattering time, *e* denotes the electron charge and *ħ* is reduced Planck’s constant. At microwave frequencies the Boltzmann equation represents an accurate approximation to the more general Kubo formula for the conductivity, because the interband contribution to *σ* is negligible for frequencies below 100 GHz^5^. In equation 3, all energies are referenced to the Dirac point energy. In our calculations, we assume an energy-independent scattering time whose value is different for positive (denoted *τ*_+_) and negative (denoted *τ*_−_) energies. The chemical potential, *μ*, is determined by the carrier density, which is controlled via the gate voltage *V*_*GS*_. For a single graphene layer, the charge density is given by *n* = *n*_0_ + *CV*_*GS*_/*e*, where *n*_0_. denotes the intrinsic charge density and *C* is the areal capacitance associated with the gate dielectric between graphene and Si. For the double layer (see Fig. 3) we obtain the conductivity of graphene stacks by adding the conductivities of the individual sheets. Note that the units for conductivity are expressed in units of (4*e*^2^/*πh*), the widely accepted minimum optical conductivity of graphene^24^.

Figure 3d,e compares the measured conductivity of a single graphene sheet on nSiO_2_ and 5 nm thick AlN respectively with the simulations based on the Boltzmann equation result (equation 3). Electron and hole scattering times of *τ*_+_ = 17 fs a *τ*_−_ = 14 fs give the best fits for nSiO_2_ and *τ*_−_ = 14.5 fs for AlN as well as *n*_0_ = 8.6 × 10^11^1/cm^2^ for the intrinsic charge density of graphene are used to obtain the fit (see Supporting information). We note that these scattering times agree well with previously reported values in the literature^9^,^25^,^26^,^27^,^28^. As apparent from both plots, a good agreement between theory and experiment is achieved.

Figure 4a shows the results of the microwave field effect characterization of a large area (25 mm^2^) double graphene-FET as a function of the applied voltage between the two graphene layers. The different curves correspond to different voltages applied to the HRS back gate. Note that the partial transparency of graphene and AlN to the electromagnetic cavity fields enables the measurements of the parallel resistance of the two graphene layers. In order to understand the variation of the sheet resistance with the voltage applied to the Si back-gate and the measured asymmetry of the curves, simulations based on the Boltzmann equation were performed. Figure 4b and c compare the experimental conductivity results for the graphene stack with results of simulations. In our calculations, we used *τ*_+_ = *τ*_−_ = 3.6 fs and *n*_0_ = 10.8 × 10^11^1/cm^2^ for the top graphene sheet and *τ*_+_ = 10.9 fs, *τ*_−_= 5.8 fs and *n*_0_ = −4.0 × 10^11^1/*c*m^2^ for the bottom graphene sheet. Areal capacitances of 0.3 mF/m^2^ and 0.17 mF/m^2^ were used for the top capacitor (50 nm AlN) and the bottom capacitor (nSiO_2_), respectively.

The simulations reproduce some of the key features of the experimental result, including the shift of the Dirac point of the bottom graphene layer for changes of the back gate voltage and the strong asymmetry of the conductivity curves, which arises from the presence of the top graphene layer whose Dirac point is at a large negative bias voltage which lies outside the range of leakage-free voltages between the two graphene layers. It is important to note that the induced current in the bottom graphene layer is slightly higher than the current in the top graphene layer due to partial reflection of the magnetic field from the bottom graphene layer. The results demonstrate a switching ratio as high as 4.5 for the parallel resistance due to alteration of the gate voltage between the two graphene layers, i.e. without any change of the back-gate voltage and without any metallic top gate. As such, this structure represents an all-graphene voltage-controlled attenuator for microwave frequencies. The attenuation of the microwave signal results from an Ohmic current through the graphene layers which is induced by the electromagnetic wave. Any variation of the sheet-resistance changes the amount of partial reflection and transmission. As a result, the on-off ratio of the sheet resistance is directly proportional to the on-off ratio of the attenuator. Moreover, we find that the minimum conductivity in the theoretical results increases upon increasing the back gate voltage, while its value is almost constant in the experimental measurement. We attribute this discrepancy to the assumption of a constant scattering time in the computed conductivity, which cannot reproduce the experimentally observed saturation of the conductivity at large gate voltages. Furthermore, the spacing between the resistance curves in Fig. 4b is not as regular as predicted by theory because of the piezoelectricity of AlN, which results in a voltage-dependent capacitance^29^.

In conclusion, we fabricated an all graphene field-effect device that operates at microwave frequencies and established a microwave cavity technique as a powerful tool for the characterization of such a field effect device. A structure composed of a 50 nm thick aluminum nitride layer between two graphene layers exhibits variation of the stack sheet resistance with a pronounced Dirac point and an on-off ratio of 4.5 by applying a DC voltage between the two graphene layers only. Use of native oxide gate dielectrics in combination with annealing at moderate temperatures results in reproducible transfer curves with charge neutrality points around zero volts, which are in very good agreement with the expectation from our theoretical model. This structure possesses a great potential for microwave-to-THz applications, such as in voltage-controlled attenuators and modulators for high data-rate wireless communication. Moreover, this technology may be used to build integrated electrodes for piezoelectric devices enabling excitation of mechanical oscillations in AlN based thin film bulk acoustic devices (FBAR) and thin film microbridges at RF and microwave frequencies. It is expected that the outstanding mechanical performance of AlN is not negatively affected by the presence of ultrathin graphene layers, which possess outstanding mechanical properties themselves. The possible combination of electric biosensing by monitoring conductivity changes of functionalized graphene and mass detection by an AlN microbridge at the same time within one device can pave the way to a new generation of silicon-integrated dual-mode biosensors with two orthogonal sensor channels.

## Methods

### Graphene Growth

Large area single layer graphene was fabricated using a vertical CVD technique and transferred *via* a PMMA method, which has been described in our previous work^30^. The vertical CVD module is one of the core parts of the cluster PVD-CVD deposition equipment (Supporting information S1). In this work single-layer graphene films are grown on copper foils (35 μm thick and 99.95% purity foils from Graphene Platform Corporation) at a pressure of 2Torr under the flow of hydrogen and methane with H_2_/CH_4_ gas flow ratio of 4. The growth temperature is set to 1050 °C for the duration of 30 minutes. In fabrication of the DG-FET structures, graphene films were grown on a 7 mm by 5 mm copper substrates to facilitate the transfer process.

### Microwave Setup

**Figure 1:**
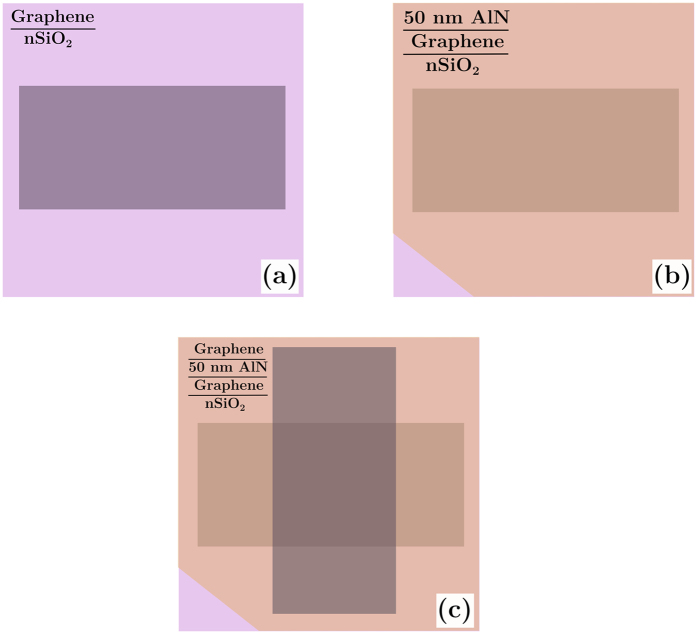
Step by step fabrication procedure of the double-graphene FET on a silicon substrate with native silicon dioxide on its surface. The transfer of the first layer of graphene (**a**) is followed by deposition of 50 nm of AlN over the surface (**b**). The device manufacturing is completed by transferring of the second graphene layer at a 90° rotation with respect to the first graphene layer.

A 3 mm thick cylindrical dielectric puck (made of barium zinc tantalate (BZT)) with a diameter of 7 mm, a very low loss-tangent at microwave frequencies (ca. 10^−4^ at 10 GHz) and a high relative permittivity (29) is placed inside a copper cavity. The puck is fixed on top of a quartz holder approximately 1 mm below a circular aperture of 5 mm diameter, which is located at the center of the top wall of the cavity and allows the resonant field to interact with the graphene sample (Fig. 1).

### AlN Magnetron Sputtering

An AlN insulating layer was deposited using radio frequency (RF) magnetron sputtering with thickness of 50 nm. The RF sputtering process was conducted in a Mantis combined sputtering/thermal evaporation system with a base pressure of 5 × 10^−8^ mbar using a 40% N_2_/Ar partial pressure. The growth pressure was 4 × 10^−3^ mbar at 200 W and the temperature of the substrate was set to 400 °C.

#### Graphics

**Figure 2:**
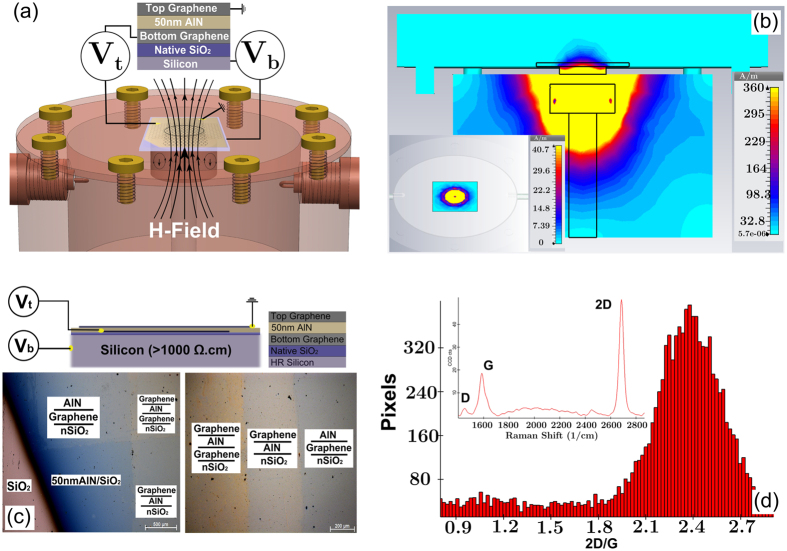
(**a**) Schematics of the microwave cavity setup for characterization of graphene-based FET structures. (**b**) Electric field simulations at around 8.7 GHz, showing the leakage of the evanescent field through the aperture as well as through the substrate and the generation of circular currents in the graphene layer. (**c**) Optical image and structure arrangement of the device, showing the contrast between different layers of the self-gating graphene structure (scales on the left/right micrographs are 500 μm/200 μm). (**d**) A typical Raman spectrum and 2D/G distribution in graphene after AlN deposition.

The schematic in Figs 1 and 2 are created using Inscape v0.91 and SolidWorks 2015 software.

## Additional Information

**How to cite this article**: Adabi, M. *et al*. Microwave Study of Field-Effect Devices Based on Graphene/Aluminum Nitride/Graphene Structures. *Sci. Rep.* **7**, 44202; doi: 10.1038/srep44202 (2017).

**Publisher's note:** Springer Nature remains neutral with regard to jurisdictional claims in published maps and institutional affiliations.

## Supplementary Material

Supplementary Information

## Figures and Tables

**Figure 3:**
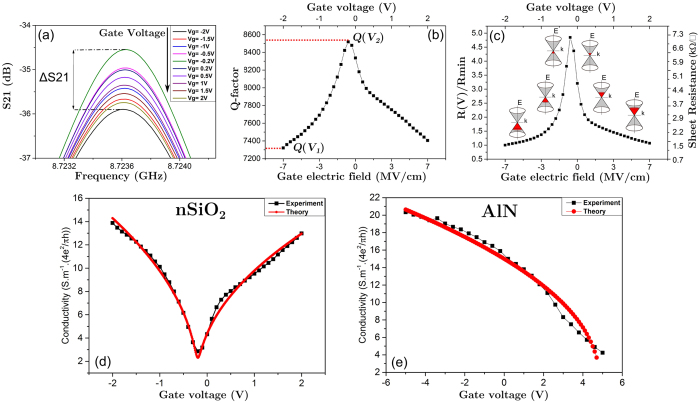
(**a**) Gate voltage dependent resonant curves that are used to extract (**b**) the Q-factor as a function of gate voltage and (**c**) sheet resistance of graphene as a function of gate voltage via equations 1 and 2. (**d**,**e**) Conductivity vs gate voltage of G-FET structures and comparison with a fit of the Boltzmann equation for Si back-gated G-FETs with nSiO_2_ and AlN as gate dielectrics, respectively.

**Figure 4:**
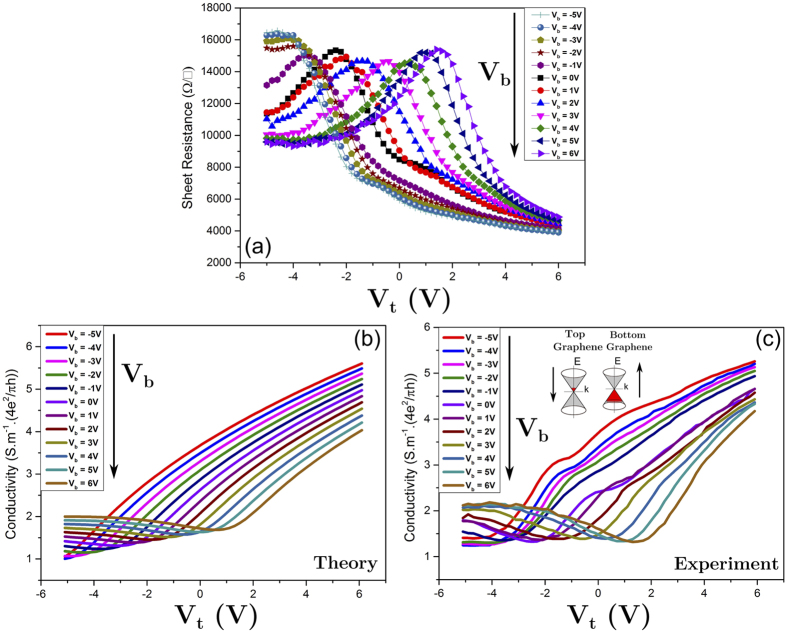
(**a**) Sheet resistance variation with respect to top and bottom gate voltages. (**b**) Theoretical and (**c**) experimental transport FET characteristics for top and bottom gate dependence graphene self-gated structure. The configuration of both bottom and top gate voltages can be seen in Fig. 2c.
