# Supplementary material for: Microwave Study of Field-Effect Devices Based on Graphene/Aluminum Nitride/Graphene Structures
Source: Sci Rep. 2017 Mar 9;7:44202. doi: 10.1038/srep44202 (PMC5343569; doi:10.1038/srep44202)
Supplement: Supplementary Information [file srep44202-s1.pdf]

Supporting Information for

Microwave Study of Field-Effect Devices Based on Graphene/Aluminum Nitride/Graphene Structures

M. Adabi[1]*, J. Lischner[1], S. M. Hanham[1], A. P. Mihai[1] O. Shaforost[1,2], R. Wang[1,2], L. Hao[2], P. Petrov[1] and N. Klein[1]

1 Department of Materials, Imperial College London, London SW7 2AZ, UK.
2 National Physical Laboratory, Teddington, Middlesex, TW11 0LW, UK.

Corresponding author: mohammad.adabi09@imperial.ac.uk

Contents

Part I: Vertical CVD graphene synthesis

S1: Photograph and schematics of the vertical CVD graphene production system

Part II: Measurement Configuration

S2: Experimental setup for DC and microwave field-effect measurements

Part III: Modelling and Simulations

S3: CST Microwave Studio simulations of the microwave assembly

S4: Electrical circuit of the bottom-gated G-FETs

S5: Conductivity calculations

Part IV: DC and Microwave Agreement

S6: Match between DC and Microwave data on different substrates

Part V: Characterisation of Graphene and AlN Thin Films

S7: AFM, XRD, and Raman spectroscopy analysis of graphene and AlN thin films

Part I: Vertical CVD graphene synthesis

S1. Photograph and schematic of the vertical CVD graphene production system

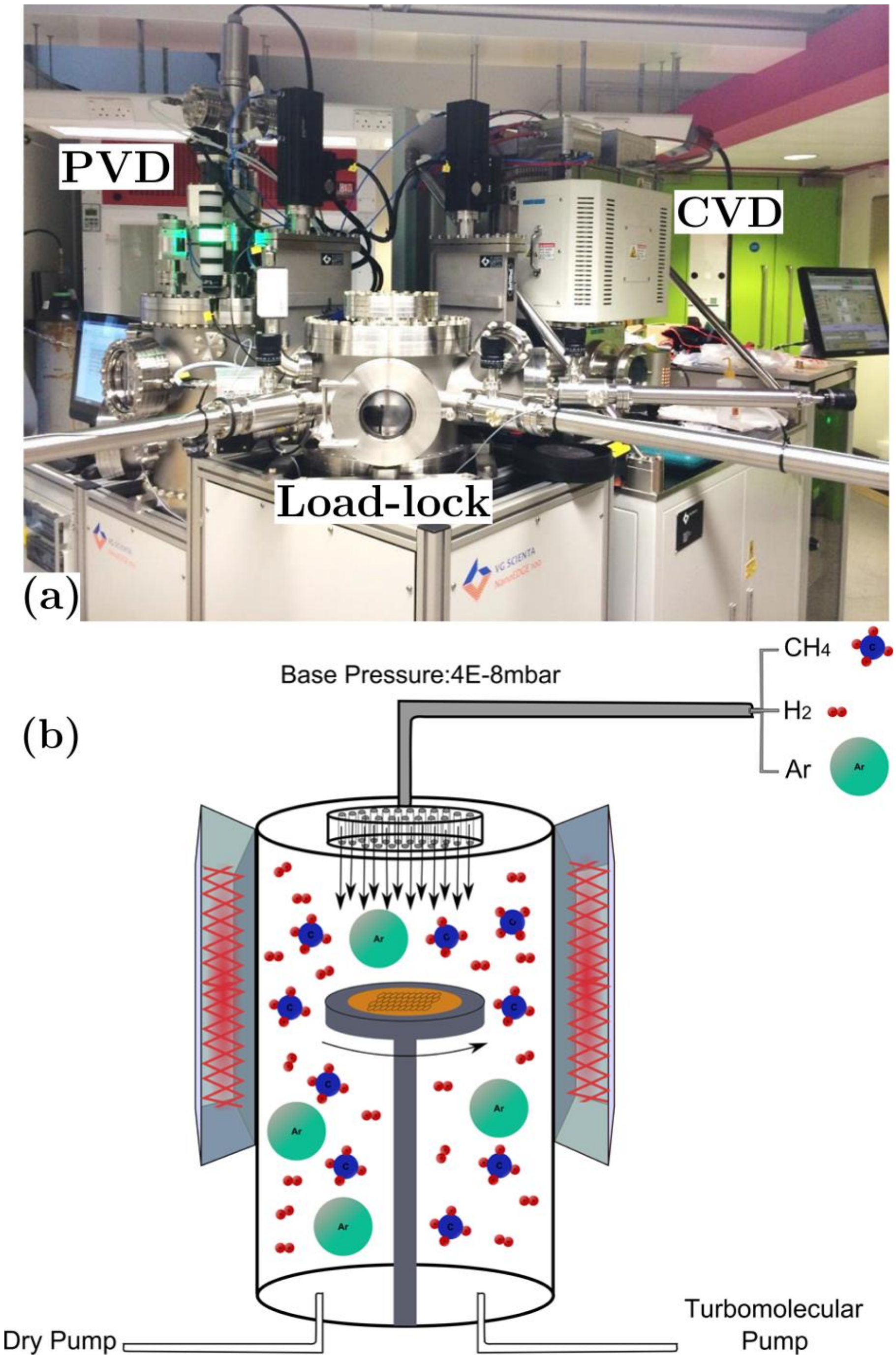

Figure 1. (a) Photograph of a cluster PVD-CVD deposition system. **(b)** Schematic of the CVD module and its components.

Part II: Measurement Configurations

S2. Experimental setup for DC and microwave field-effect measurement

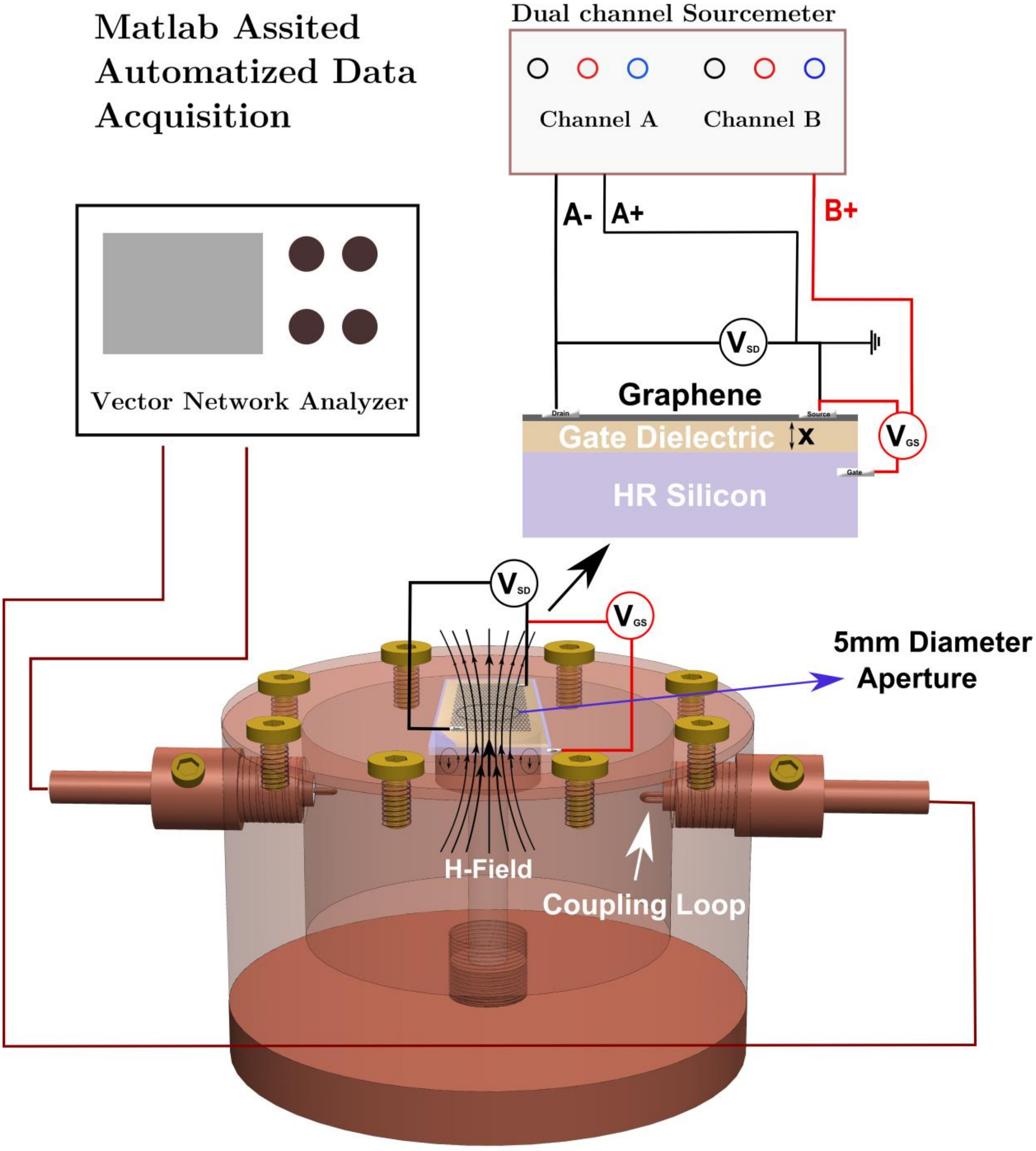

Figure 2. Matlab-based automatized data acquisition setup developed to measure DC and microwave field effect properties of fabricated devices.

Part III: Modelling and Simulations

S3. CST Microwave Studio simulations of the microwave assembly

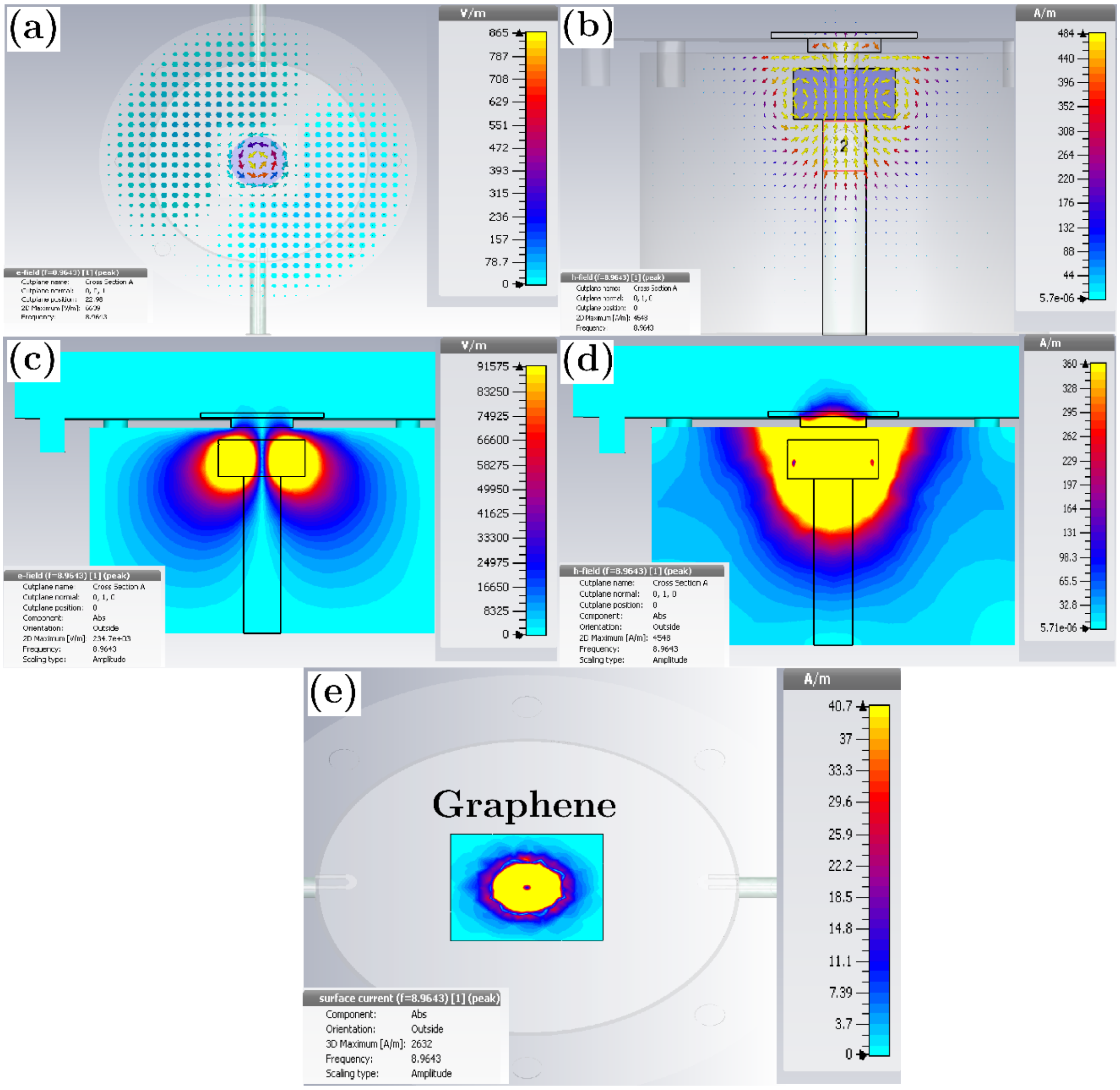

Figure 3. Frequency domain microwave simulation at resonant frequency confirming the transverse electrical nature of the chosen resonant mode. Simulations demonstrate distribution of **(a)** & **(c)** electric field, **(b)** & **(d)** magnetic field in the cavity and **(e)** graphene's surface current density.

S4. Scheme for gate biasing and dc electric measurements

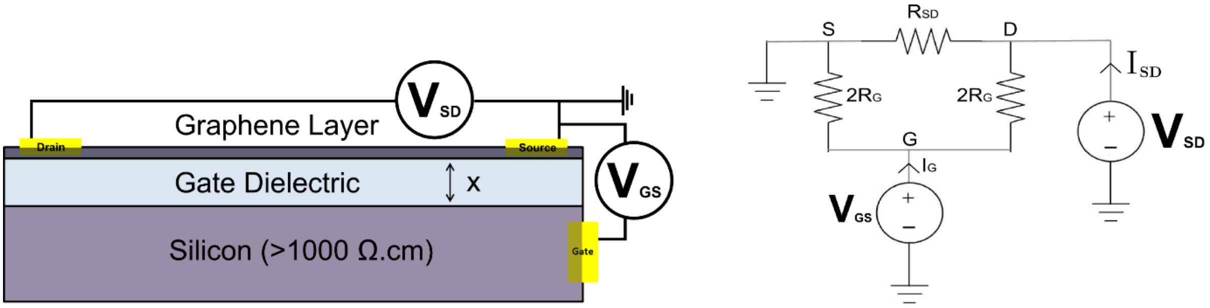

Figure 4. Wiring scheme and equivalent circuit of the back-gated G-FET structure, taking small gate leakages into account.

S5. Conductivity calculations

To obtain the conductivity of single graphene sheets and graphene stacks, we employ the Boltzmann equation. The resulting expression for the DC conductivity is equivalent to the Kubo formula with intraband transitions only. In Hartree atomic units, the result for a single graphene sheet is

$$\sigma_{\alpha\beta} = -4\sum_{n}\int \frac{d^2k}{(2\pi)^2}\frac{1}{2\Gamma(\epsilon_{nk})}v_{\alpha nk}v_{\beta nk}\frac{\partial f}{\partial \epsilon}(\epsilon_{nk}),$$

where α and β denote the Cartesian direction, $\Gamma(\epsilon)$ is the energy-dependent scattering rate, $\vec{v}_{nk} = v_F\hat{k}$ (with v_F denoting the Fermi velocity of graphene) and $\epsilon_{nk} = \pm v_F|k|$. Moreover, $f(\epsilon) = 1/(\exp\left[\frac{\epsilon-\mu}{k_BT}\right] + 1)$ is the Fermi-Dirac distribution, where μ denotes the chemical potential, T the temperature and k_B the Boltzmann constant. Also, the index n labels the two Dirac bands of graphene and we have taken valley- and spin-degeneracy into account via the factor of 4.

The angular integral can be carried out analytically resulting in

$$\sigma_{\alpha\beta} = -\delta_{\alpha\beta}v_F^2\sum_{n}\int \frac{dk}{\pi}\frac{k}{\Gamma(\epsilon_{nk})}\frac{\partial f}{\partial \epsilon}(\epsilon_{nk}) = \frac{1}{2\pi k_BT}\int_{-\infty}^{\infty} d\omega\frac{|\omega|}{\Gamma(\omega)}\frac{e^{(\omega-\mu)/k_BT}}{\left[e^{\frac{\omega-\mu}{k_BT}}+1\right]^2}.$$

In the above expression, we changed the variable of integration from k to ω and then combined the contribution from both Dirac bands into a single integral over ω.

To evaluate the above equation, the chemical potential and the scattering rate must be determined. Using the value of experimentally applied gate voltages, we first deduce the areal density of charge carriers as explained in the main section of manuscript. The charge carrier density is a sum of an electron contribution from the upper Dirac band and a hole contribution from the lower Dirac band. For a given value of the chemical potential, these contributions can be calculated via

$$n_e = \frac{2}{\pi v_F^2} \int_0^{\infty} d\omega |\omega| f(\omega),$$

$$n_h = \frac{2}{\pi v_F^2} \int_{-\infty}^{0} d\omega |\omega| (1 - f(\omega).$$

We numerically invert these equations to deduce the chemical potential from n_e and n_h. Finally, - as explained in the main section of the manuscript – we assume that the scattering rate is different for electrons in the two Dirac bands, but constant otherwise.

Part IV: DC and Microwave Agreement

S6. Match between DC and Microwave data on different substrates

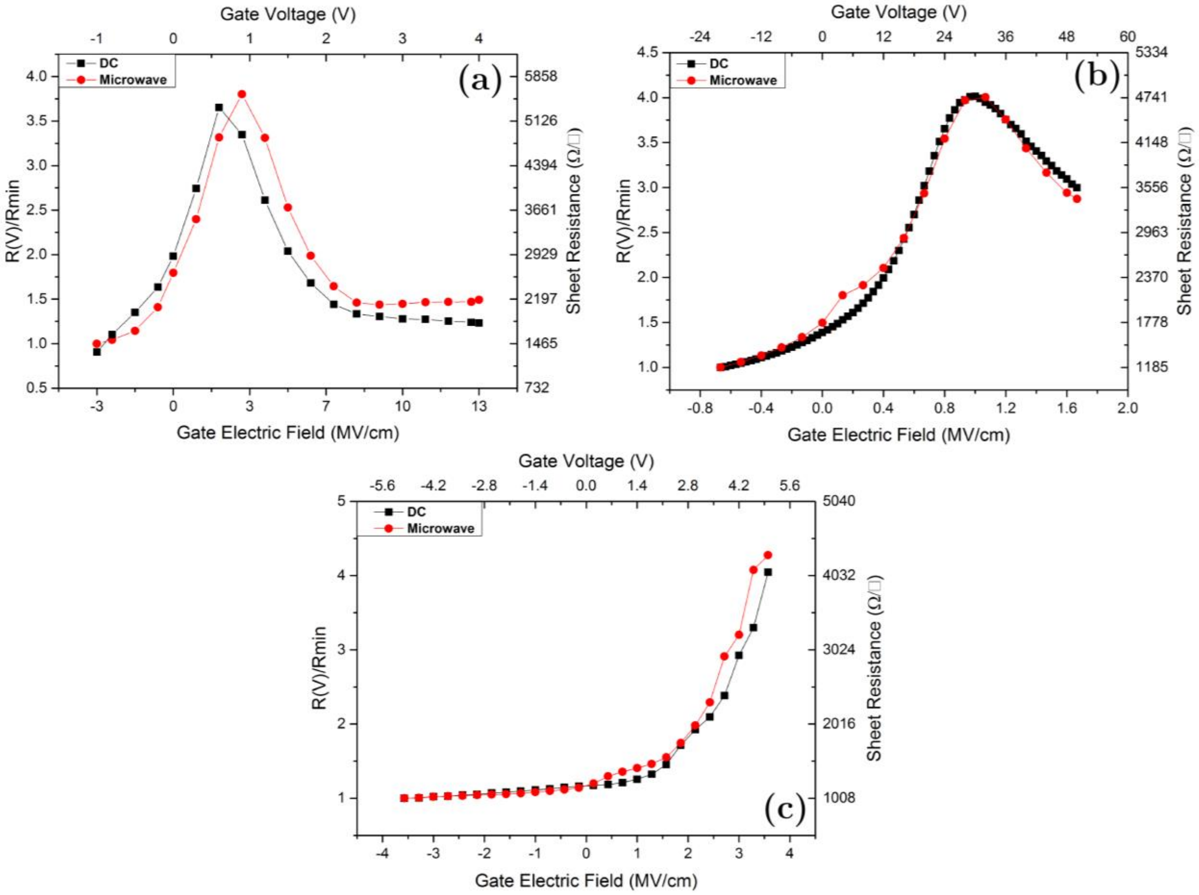

Figure 5. Comparison of microwave and DC resistance data for back-gated FET structures with **(a)** nSiO_2, **(b)** 300 nm SiO_2, and **(c)**5nm AlN gate dielectric before annealing. The good agreement is indicative of the accuracy and consistency of the method.

Part V: Characterisation of Graphene and AlN Thin Films

S7. AFM, XRD, and Raman spectroscopy analysis of graphene and AlN thin films

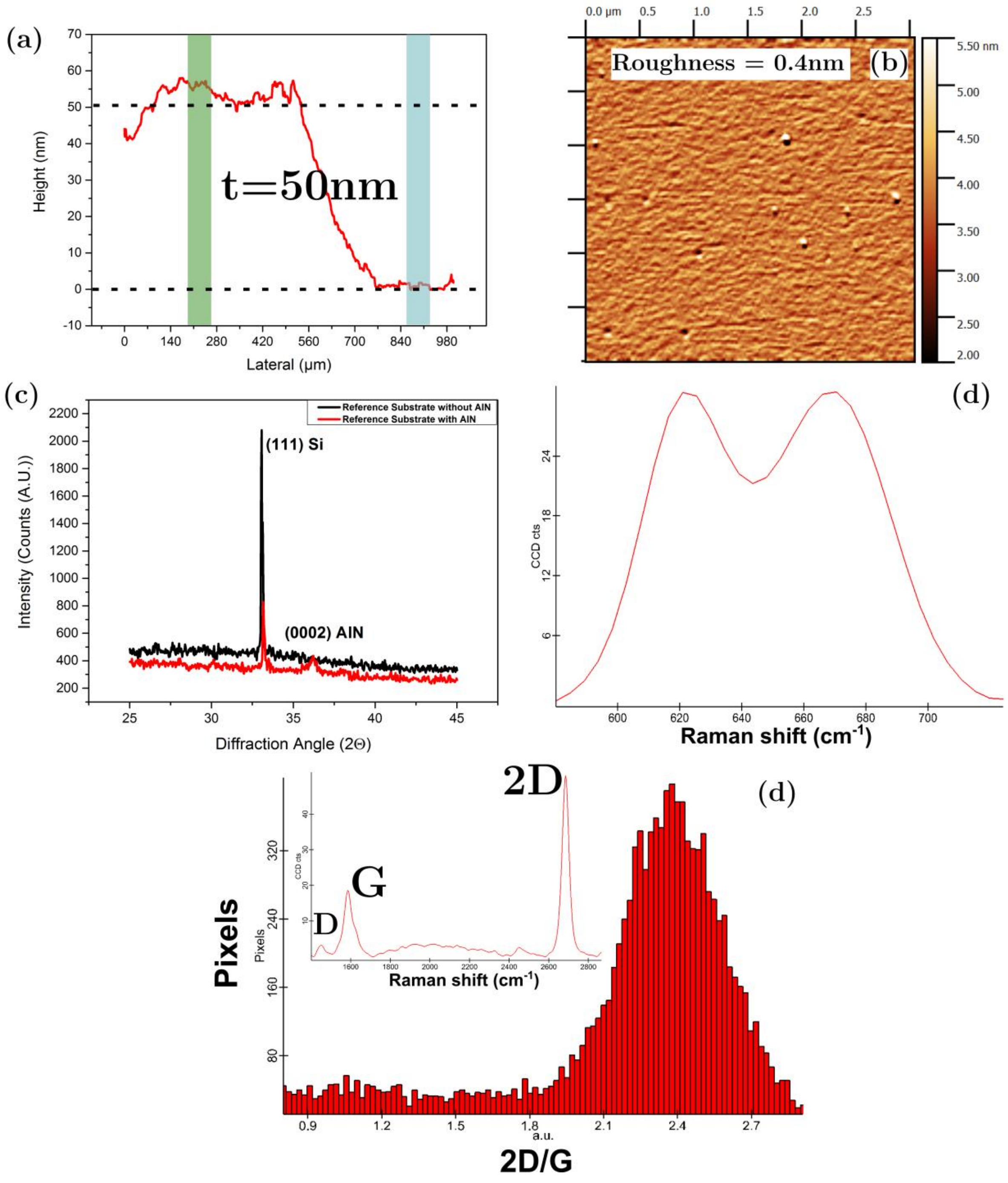

Figure 6. (a) Thickness profile of the sputtered AlN thin film indicating thickness of 50nm and **(b)** its surface roughness indicative of a smooth surface. **(c)** X-ray diffraction spectrum of the AlN film showing a (0002) plane orientation of the grown film indicating a high degree of c-axis orientation.[1] (d) Raman spectrum of crystalline AlN thin film sandwiched between the two graphene. (e) Raman characterization of graphene samples with mean 2D/G peak ratio of about 2.4 indicating the monolayer nature of our graphene films with minimal defects.

Capacitance measurements on AlN films yielded capacitance of 1.52×10^{-11} F, resulting in permittivity value of 1.7, much lower than the observed value for the bulk material. [2] This is believed to be a direct result of using a relatively low growth temperature.

References

[1].Jagannadham, K.; Sharma, A. K.; Wei, Q.; Kalyanraman, R.; Narayan, J., Structural characteristics of AlN films deposited by pulsed laser deposition and reactive magnetron sputtering: A comparative study. *JOURNAL OF VACUUM SCIENCE AND TECHNOLOGY A VACUUMS SURFACES AND FILMS* **1998,** *16* (5), 2804-2815.
[2].Lenie, C.; Carborundum Co, N. F. N. Y., SOME PROPERTIES OF ALUMINUM NITRIDE. *Journal of the Electrochemical Society (U.S.) Absorbed Electrochem. Technol.* **1960,** *Vol: 107*, 308-14.
